# Sweet potato NAC transcription factor *NAC43* negatively regulates plant growth by causing leaf curling and reducing photosynthetic efficiency

**DOI:** 10.3389/fpls.2023.1095977

**Published:** 2023-02-21

**Authors:** Sifan Sun, Xu Li, Nan Nie, Yanqi Chen, Shaopei Gao, Huan Zhang, Shaozhen He, Qingchang Liu, Hong Zhai

**Affiliations:** Key Laboratory of Sweet Potato Biology and Biotechnology, Ministry of Agriculture and Rural Affairs/Beijing Key Laboratory of Crop Genetic Improvement/Laboratory of Crop Heterosis and Utilization, Ministry of Education, College of Agronomy and Biotechnology, China Agricultural University, Beijing, China

**Keywords:** sweet potato, *IbNAC43*, plant growth, leaf curling, photosynthetic rate

## Abstract

Leaves comprise one of the most important organs for plant growth and development. Although there have been some reports on leaf development and the establishment of leaf polarity, their regulatory mechanisms are not very clear. In this study, we isolated a NAC (NAM, ATAF, and CUC) transcription factor (TF), i.e., *IbNAC43*, from *Ipomoea trifida*, which is a wild ancestor of sweet potato. This TF was highly expressed in the leaves and encoded a nuclear localization protein. The overexpression of *IbNAC43* caused leaf curling and inhibited the growth and development of transgenic sweet potato plants. The chlorophyll content and photosynthetic rate in transgenic sweet potato plants were significantly lower than those in wild-type (WT) plants. Scanning electron microscopy (SEM) and paraffin sections showed that the ratio of cells in the upper and lower epidermis of the transgenic plant leaves was unbalanced; moreover, the abaxial epidermal cells were irregular and uneven in transgenic plants. In addition, the xylem of transgenic plants was more developed than that of WT plants, while their lignin and cellulose contents were significantly higher than those of WT. Quantitative real-time PCR (qRT-PCR) analysis showed that the overexpression of *IbNAC43* upregulated the genes involved in leaf polarity development and lignin biosynthesis in transgenic plants. Moreover, it was found that IbNAC43 could directly activate the expression of the leaf adaxial polarity-related genes *IbREV* and *IbAS1* by binding to their promoters. These results indicate that *IbNAC43* might play a critical role in plant growth by affecting the establishment of leaf adaxial polarity. This study provides new insights regarding leaf development.

## Introduction

Plant yield and quality cannot be achieved without normal growth and development (Gong et al., 2020). Leaves comprise one of the most important plant organs where CO_2_ is fixed to produce carbohydrates through photosynthesis (Guo and Gan, 2014). The morphology of leaves is an important aspect of plant architecture that affects light interception, water loss, photosynthesis, and respiration (Otoole and Cruz, 1980; Hou et al., 2020). Moderate leaf curling helps increase the light-receiving space and improves the utilization rate of light energy, thereby increasing the yield of plants (Xu et al., 2018). Leaf curling also enhances drought tolerance by reducing water loss (Li et al., 2017). However, severe leaf curling often leads to growth retardation and developmental defects (Yuan et al., 2015). The shape of the leaves generally depends on the establishment of leaf polarity, in which adaxial–abaxial polarity plays an important role in leaf curling (Manuela and Xu, 2020) and is regulated by complex molecular mechanisms. Therefore, understanding the established mechanisms of leaf adaxial–abaxial polarity is extremely important for improving plant growth and resistance to abiotic stress.

Recently, genetic studies have reported that different genes affect leaf curling in various species, such as in *Arabidopsis thaliana* (Husbands et al., 2015), tomato (Pulungan et al., 2018), soybean (Yang et al., 2019), and rice (Sun et al., 2020; Xu et al., 2021). Leaf curling can be differentiated into two types based on the classification of plants, i.e., monocotyledons and dicotyledons. In monocotyledonous plants, such as rice, the phenotype of leaf curling, in general, is caused by the change of bulliform cells (Li et al., 2010; Fang et al., 2012; Li et al., 2016; Sun et al., 2020; Xu et al., 2021). On the other hand, leaf curling in dicotyledonous plants is mainly related to the adaxial–abaxial leaf polarity establishment, cutin and cuticular wax biosynthesis, and hormone responses (Wan et al., 2021). The first gene associated with leaf polarity, *phantastica* (*PHAN*), was required for the elaboration of the proximodistal axis (Waites and Hudson, 1995; Waites et al., 1998). Since then, extensive evidence has indicated that the regulation pathway of leaf polarity is extremely complex and that many leaf development-associated genes are involved in regulatory processes. The members of the *HD-bZIP* III family—*REVOLUTA* (*REV*), *PHABULOSA* (*PHB*), and *PHAVOLUTA* (*PHV*)—are involved in the control of leaf adaxial cells (McConnell et al., 2001; Otsuga et al., 2001). In addition, ASYMMETRIC LEAVES1/AtMYB91 (AS1) physically interacts with ASYMMETRIC LEAVES2 (AS2) to maintain the normal development of leaves together with the *trans*-acting small interfering RNA (ta-siRNA) pathway (Iwakawa et al., 2007; Husbands et al., 2015; Iwakawa et al., 2020). On the other hand, the members of the YABBY family—*AtYAB3/FIL*, *AtYAB5*, *BcYAB3*, and *GmFILa*—regulate the fate of leaf abaxial cells (Sawa et al., 1999; Siegfried et al., 1999; Yang et al., 2019; Hou et al., 2020). At present, there are only a few reports on NAC (NAM, ATAF, and CUC) transcription factors (TFs) involved in the regulation of leaf curling.

The NAC family is one of the important TF families in plants, and NACs play important roles in plant growth, development, and abiotic and biotic stress responses (Puranik et al., 2012; Nuruzzaman et al., 2013; Kim et al., 2016). Genetic studies have revealed that most NACs, including *ANAC092/ORESARA1* (*AtORE1*), *ANAC029*/*Arabidopsis* NAC-LIKE Activated by AP3/PI (*AtNAP*), *ANAC059*/*ORESARA1 SISTER1* (*AtORS1*), *AtATAF2*, *AtANAC016*, and *NaNAC29*, as positive regulators, and *ANAC042/JUNGBRUNNEN1* (*AtJUB1*) and *ANAC083/VND-INTERACTING2* (*AtVNI2*), as negative regulators, are mainly involved in the control of leaf senescence (Balazadeh et al., 2011; Yang et al., 2011; Wu et al., 2012; Kim et al., 2013; Kim et al., 2016; Nagahage et al., 2020; Ma et al., 2021). Furthermore, the NAC TFs *CUC1*, *CUC2*, and *CUC3* specify the formation of leaf boundary (Vroemen et al., 2003; Laufs et al., 2004; Hibara et al., 2006). At present, there is no report on the function of *NAC43* in plants.

Sweet potato is a globally important food crop known for its strong adaptability, abundant nutrient content, high yield, and diverse uses (Ahn et al., 2010). In general, the morphology and photosynthetic activity of leaves can affect plant growth and crop yield (Zhu et al., 2010). Although it is important to understand the mechanism of leaf polarity development, there have been no reports on the leaf development-related genes in sweet potato plants, particularly the leaf curling-related genes. In this study, we isolated the NAC TF *IbNAC43* from *Ipomoea trifida*, which is a wild ancestor of sweet potato. The overexpression of this TF caused leaf curling, reduced the photosynthetic rate, and retarded growth in transgenic sweet potato. These results indicate that *IbNAC43* plays an important role in leaf polarity development and plant growth.

## Materials and methods

### Plant materials and growth conditions

*I. trifida* was used to isolate the *IbNAC43* gene. The sweet potato cultivar Shangshu 19 was employed to analyze the function of *IbNAC43*. *In vitro*-grown transgenic sweet potato plants (Shangshu 19) were cultured on Murashige and Skoog (MS) medium at 27 ± 1°C under a photoperiod consisting of 13 h of cool white fluorescent light at 54 μmol m^−2^ s^−1^ and 11 h darkness. Subsequently, the sweet potatoplants were cultivated in a field or glasshouse (25 ± 3°C, natural illumination) at the campus of China Agricultural University, Beijing, China.

### Cloning and sequence analysis of *IbNAC43*

Total RNA of sweet potato plants was extracted using the RNAprep Pure Plant Kit (Tiangen Biotech, Beijing, China), and first-strand complementary DNA (cDNA) synthesis was performed using PrimeScript™ II 1^st^ Strand cDNA Synthesis Kit (TaKaRa, Beijing, China). Primer pairs (*IbNAC43*-ORF-F/R) were used to amplify the full-length cDNA of *IbNAC43* (**Supplementary Table S1**). The protein of IbNAC43 was analyzed using online BLAST (TBLASTX, RRID : SCR_011823; https://blast.ncbi.nlm.nih.gov/Blast.cgi). The DNAMAN software (LynnonBiosoft, San Ramon, CA, USA) was utilized to align the amino acid (aa) sequence of IbNAC43 with those of the NAC43 proteins from different plant species. A phylogenetic tree was constructed using MEGA 5.0 software (downloaded from https://megasoftware.net/) with the neighbor-joining method, which had 1,000 bootstrap replications (Kumar et al., 2018).

### Expression analysis of *IbNAC43* in sweet potato

The transcript levels of *IbNAC43* in the roots, stems, and leaves were measured with Shangshu 19 using quantitative real-time PCR (qRT-PCR). The expression levels were normalized to those of *Ibactin* (AY905538). The related qRT-PCR primer pairs are listed in **Supplementary Table S1**.

### Subcellular localization of *IbNAC43*

The *IbNAC43* open reading frame (ORF) without a stop codon, amplified with a primer pair (1300-*IbNAC43*-F/R) (**Supplementary Table S1**), was ligated into pCAMBIA1300-GFP. The recombinant vectors IbNAC43-GFP, NLR-RFP (a nuclear localization marker), and 35S:GFP were transformed into the *Agrobacterium tumefaciens* strain EHA105 using the heat shock method. The translational fusion construct and NLS-RFP were transiently expressed in *Nicotiana benthamiana* leaf epidermal cells *via* infiltration of *A. tumefaciens* (Verweij et al., 2008). The construct vectors 35S:IbNAC43-GPF and 35S:GFP were transformed into rice protoplasts isolated from etiolated shoots *via* treatment with polyethylene glycol (Du et al., 2021; Sun et al., 2022). Subsequently, the green and red fluorescent protein (GFP and RFP, respectively) signals were collected using confocal laser scanning microscopy (LSM880; Zeiss, Oberkochen, Germany).

### Transactivation activity assay

Full-length IbNAC43 and 1–167, 167–372, 1–201, 1–235, 1–269, and 1–303 aa of the IbNAC43 coding sequence were amplified by PCR using different primer pairs (**Supplementary Table S1**) and inserted into the *Nde*I/*Eco*RI-digested pGBKT7 vector (Oebiotech; https://www.oebiotech.com/) to produce the fusion constructs. The fusion plasmids, a negative control (pGBKT7-empty), and a positive control (pGAL4) were separately transformed into the yeast strain AH109. The transformed yeast was streaked on SD/-Trp and SD/-Trp/-His/X-α-Gal plates for two-three days at 30°C to observe yeast growth.

### Production of transgenic sweet potato plants

The expression vector pCAMBIA1300-*IbNAC43*, containing the *IbNAC43* sequence, was controlled by the CaMV 35S promoter and an NOS terminator. The recombinant plasmids were then transformed into EHA105. Embryogenic suspension cultures of Shangshu 19 were prepared as described by Liu et al. (2001). Transformation and plant regeneration were performed with *A. tumefaciens*-mediated transformation, as previously described by Zhai et al. (2016) and Sun et al. (2022). Putative transgenic sweet potato plants were identified by PCR and qRT-PCR analyses with the primer pairs 35s-F/JD-*IbNAC43*-R and qRT-*IbNAC43*-F/R (**Supplementary Table S1**). The transgenic lines and wild-type (WT) plants were transferred in soils in a greenhouse and then grown in a field for phenotypic observation.

### Phenotype identification of transgenic plants

The leaf rolling index (LRI) of the WT and transgenic plants was measured after two and eight weeks in the greenhouse. These measurements are consistent with the method of Sun et al. (2020), as follows: the leaf blade was stretched out, and its maximum width (*L*_w_) calculated. At the same site, the natural distance of the leaf blade margins (*L*_n_) was measured. The LRI was calculated as follows: LRI (%) = (*L*_w_ − *L*_n_)/*L*_w_ × 100.

### Scanning electron microscopy

Transgenic and WT plants were grown in a greenhouse for four weeks, and then the features of the adaxial and abaxial surfaces in the same regions were observed during the mature leaf stage. Leaves were immediately fixed with 2.5% (*v*/*v*) glutaraldehyde for 2.5 h. After rinsing three times with 0.1 mol L^−1^ phosphoric acid buffer, the leaves were fixed in 1% (*v*/*v*) osmic acid for 2 h, followed by dehydration with the LEICA EM CPD 300 (Leica, Wetzlar, Germany) critical point dryer. EIKO IB-3 (Variable Electron Microscope, S3400N; Hitachi, Tokyo, Japan) was used to sputter gold on each sample, and surface images of the leaves were collected using a scanning electron microscope.

### Leaf and stem paraffin sections

For the paraffin sections, fully unfolded leaves and stems at the same position were detached from the transgenic and WT plants grown in the greenhouse, fixed with FAA [5% (*v*/*v*) glacial acetic acid, 5% (*v*/*v*) formalin, and 50% (*v*/*v*) ethanol] solution, and evacuated for 30 min with a vacuum pump. The fixed materials were then dehydrated with gradient ethanol (50%, 70%, 80%, 90%, 95%, and 100%, *v*/*v*) and gradient xylene (25%, 50%, 75%, and 100% xylene in ethanol, *v*/*v*). Subsequently, the samples were soaked in paraffin at 60°C for three days (the paraffin solution was replaced three times during this period), after which they were embedded in a paraffin block. Approximately 8-μm sections were obtained using a microtome (LEICA RM2265, Leica, Germany), dewaxed in xylene, and then stained with safranin and Fast Green FCF. Finally, the images of these sections were observed using an Echo Revolve light microscope (ECHO, San Diego, CA, USA).

### Lignin content measurement

The methods for the measurement of the lignin content followed those of Suzhou Comin Biotechnology Co., Ltd. (http://www.cominbio.com/a/shijihe/shenghuashiji/qitaxilie/2016/0502/969.html). The main steps were as follows: 1) for sample preparation, the leaves and stems were dried at 80°C to a constant weight, crushed, and weighed to about 5 mg; 2) in accordance with the instructions in the Suzhou Comin Lignin Kit (MZS-2-G), various reagents were added in sequence, and the supernatant was taken for examination; and 3) for sample determination, a UV–visible spectrophotometer was used to measure the absorbance at 280 nm.

### Cellulose content measurement

Similarly, the cellulose content was measured according to Suzhou Comin Biotechnology Co., Ltd. (http://www.cominbio.com/a/shijihe/shenghuashiji/tangdaixiexilie/2016/0324/843.html). The main steps were as follows: 1) for sample preparation, the leaves and stems were dried at 80°C to a constant weight, crushed, and weighed to about 10 mg; 2) according to the instructions in the Suzhou Comin Cellulose Kit (CLL-1-Y), various reagents were added in sequence and the supernatant taken for examination; and 3) for sample determination, a UV–visible spectrophotometer was used to measure the absorbance at 620 nm.

### qRT-PCR analysis

Transgenic and WT plants were grown in a greenhouse for four weeks and their leaves were used to measure the expression of the leaf polarity-related genes. qRT-PCR was performed to quantify the messenger RNA (mRNA) expression levels. qRT-PCR was carried out using a QuantStudio 6 Flex (Applied Biosystems by Thermo Fisher Scientific, Waltham, MA, USA) with the SYBR Fast Universal qPCR Kit (Forscience, Beijing, China). The primers used for qRT-PCR are listed in **Supplementary Table S1**. Relative gene expression was quantified using the ΔΔ*C*_T_ method (Pfaffl, 2001).

### Chromatin immunoprecipitation assay

The chromatin immunoprecipitation (ChIP) assay was accomplished as previously described (Bowler et al., 2004; Zheng et al., 2021). Briefly, fresh *in vitro*-grown materials were collected and subjected to vacuum infiltration in 1% (*v*/*v*) formaldehyde for 15 min to crosslink the chromatin proteins to the DNA. Fixation was stopped by adding glycine to a final concentration of 0.125 M. The fixed samples were washed three times with ddH_2_O and ground to a powder in liquid nitrogen. Chromatin was extracted and sonicated using an Ultrasonic Homogenizer (SCIENTZ, JY 92-IIDN, Ningbo, China). The anti-GFP antibody was purchased from Abmart Shanghai Co., Ltd. (M20004M, Shanghai, China). The immunoprecipitated DNA was then analyzed with quantitative PCR (qPCR), and the amplified DNA from the chromatin fractions prior to antibody incubation was used as the control. PCR reactions were performed in triplicate for each sample, and the enrichments were normalized to the input sample. The *β*-*actin* gene was used as an endogenous control. The gene-specific primers used for ChIP–qPCR are listed in **Supplementary Table S1**.

### Electrophoretic mobility shift assay

The full-length *IbNAC43* cDNA was amplified with specific primers and fused into the *Bam*HI and *Eco*RI sites of the expression vector pET-28a (Novagen; https://www.merckmillipore.com/CN/zh). The construct vector was introduced into *Escherichia coli* BL21 (DE3) cells to produce recombinant 6×His-IbNAC43 proteins induced by 0.03 mM isopropylthio-β-galactoside (IPTG) and then grown at 16°C. The 6×His-IbNAC43 protein was purified with Ni-NTA Agarose (Forscience, Beijing, China) as described previously (Zhang et al., 2022). Labeled probes with biotin at their 5′-ends were used as binding probes, while unlabeled probes were used as competitors. The primer and probe sequences are listed in **Supplementary Table S1**. The electrophoretic mobility shift assay (EMSA) was performed using a LightShift Chemiluminescent EMSA Kit (Thermo Fisher, Waltham, MA, USA) according to the manufacturer’s instructions.

### Dual-luciferase assay

The coding sequence of *IbNAC43* was cloned into the *Pst*I and *Kpn*I sites of the pGreenII 62-SK vector, which was used as an effector. The empty pGreenII 62-SK vector was used as a negative control. The *IbREV* and *IbAS1* promoters (*Kpn*I and *Pst*I sites) were inserted upstream of luciferase (LUC) into the pGreenII 0800-LUC vector to generate the *IbREVpro : LUC* and *IbAS1pro:LUC* reporter constructs. Rice protoplasts were isolated and used for dual-LUC assays as described previously (Zhang et al., 2020). The activity levels of firefly and Renilla (REN) LUC were measured using the Dual-Luciferase Reporter Assay System (Promega, Madison, WI, USA). LUC activity was normalized to REN activity. The specific primers used for the dual-LUC assay are listed in **Supplementary Table S1**. Three biological replicates were performed for this analysis.

### Y1H assay

For the yeast one-hybrid (Y1H) assay, the partial *IbCCoAoMT* and *IbPAL* promoter sequences were cloned, recombined with the pAbAi vector, and then integrated with the genome of the Y1H-Gold yeast strain. The ORF of *IbNAC43* was amplified, cloned into the pGADT7 vector, and transferred into the bait yeast strain. The detailed process was in accordance with the Yeast One-Hybrid System—Matchmaker Gold Kit (catalog no. 630491; Clontech, Mountain View, CA, USA). The co-transformed yeast cells were cultured on the SD/-Leu plate with or without aureobasidin A (AbA) at 30°C for 48 h.

### Statistical analysis

The values are presented as the mean ± SD. A Student’s *t*-test was used to examine significant differences. Significance levels were determined at *p < 0.05 and **p < 0.01.

## Results

### Cloning and sequence analysis of *IbNAC43* and its promoter

**Figure 1:**
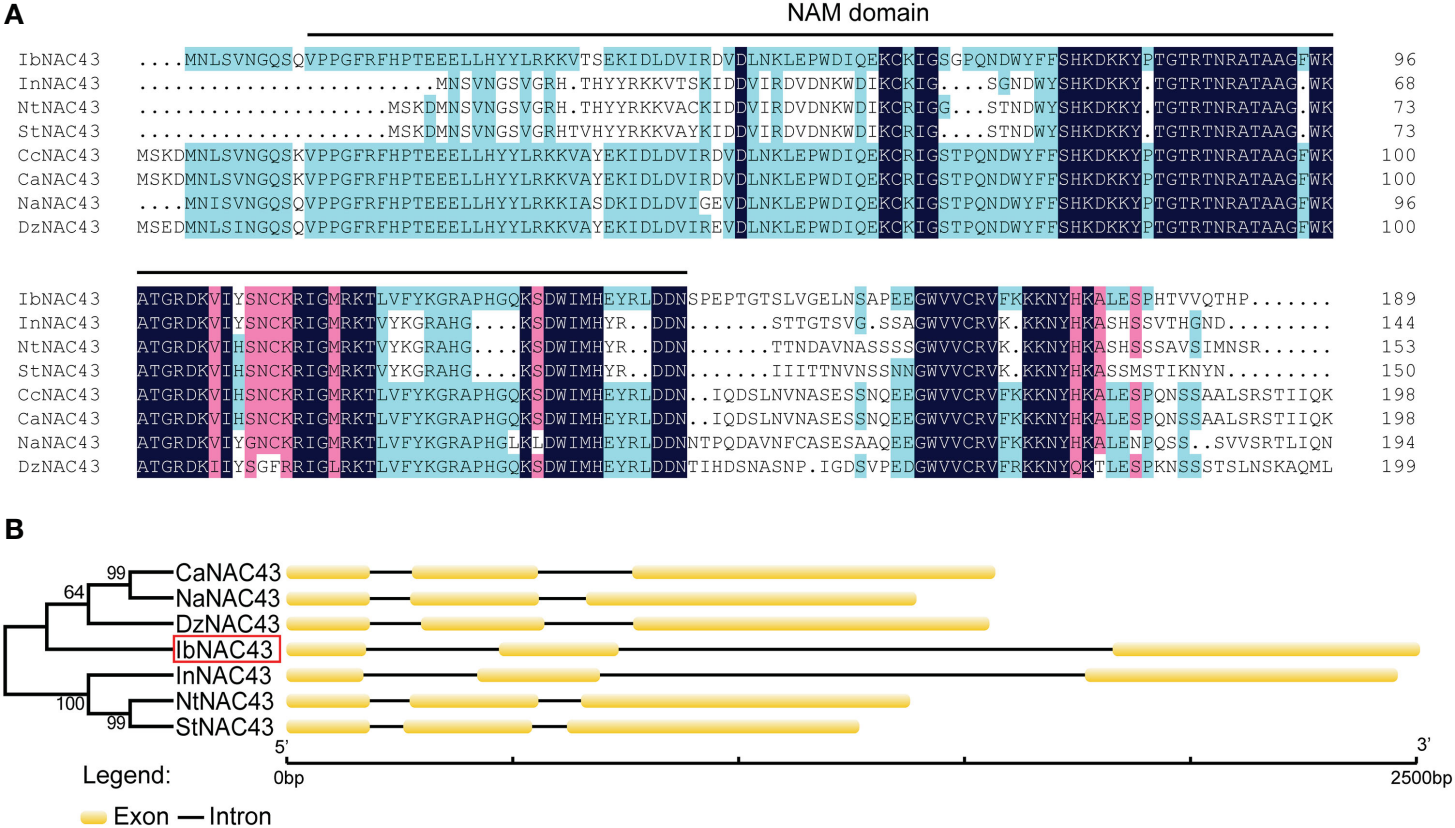
Sequence analysis of IbNAC43. **(A)** Multiple sequence alignment of the bNAC43 protein and its closest homologs from different plant species. The NAM (no apical meristem) domain is represented by *black lines*. **(B)** Phylogenetic relationships and genomic structures of *IbNAC43* and its closest homologs from different plant species. The *IbNAC43* cloned in this study is indicated by a *red box*. Exons are represented by *boxes*, while introns are represented by *lines*.

The *IbNAC43* cDNA sequence was 1,492 bp in length and contained a 1,119 bp ORF encoding a 372-aa protein with a predicted molecular weight of 41.78 kDa, and the IbNAC43 protein had a conserved NAM (no apical meristem) domain. The genomic sequence of *IbNAC43* was 2,501 bp in length and contained three exons and two introns (**Figure 1**). To elaborate on the difference in *NAC43* between the WT and cultivated sweet potato, Shangshu 19 was also used to isolate the *IbNAC43* gene. The protein of NAC43 in *I. trifida* showed a high degree of sequence identity with that in Shangshu 19 (98.92%), and the genomic sequence of *IbNAC43* in Shangshu 19 also contained three exons and two introns (**Supplementary Figure S1**). Phylogenetic analysis showed that *IbNAC43* had a close relationship with *Durio zibethinus* (XP_022773628.1, 55.07%), *Nicotiana attenuata* (XP_019229017.1, 50.53%), and *Ipomoea nil* (XP_019168629.1, 47.58%) (**Figure 1**). A 1,618-bp fragment corresponding to the promoter of *IbNAC43* was cloned from *I. trifida*, and this promoter region contained numerous types of *cis*-acting elements, such as the abscisic acid-responsive element (ABRE), G-box, gibberellin-responsive element (GARE)-motif, MYB, and MYC, which are associated with different types of stresses (**Supplementary Table S2**).

### Expression of *IbNAC43* in different issues

**Figure 2:**
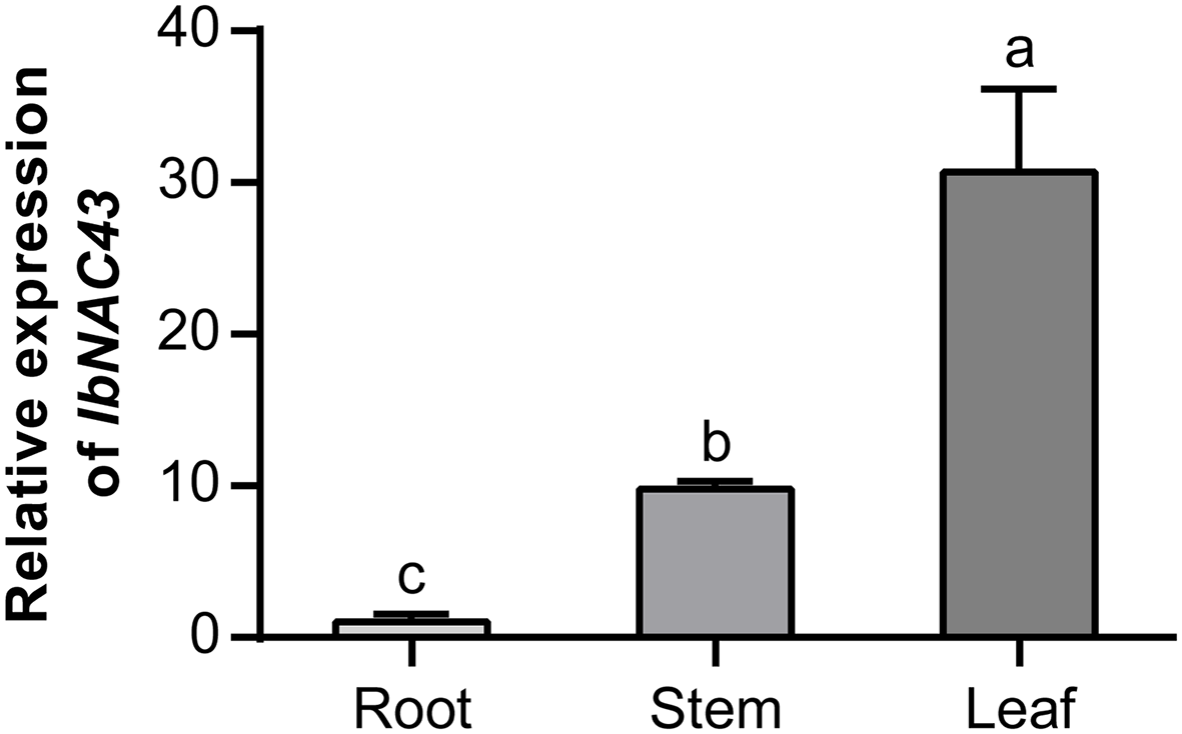
Analysis of the expression of *IbNAC43* in the root, stem, and leaf tissues of Shangshu 19. Data are presented as the mean ± SE (*n* = 3). *Different lowercase letters* indicate significant differences (*p* < 0.05, one-way ANOVA).

A qRT-PCR analysis was performed to examine the expression level of *IbNAC43* in sweet potato. The results showed that *IbNAC43* was more highly expressed in the leaves and stems than in the roots (**Figure 2**). This result indicates that *IbNAC43* might be involved in the development and function of leaves.

### IbNAC43 is a nuclear localization protein and has self-activation in yeast cells

**Figure 3:**
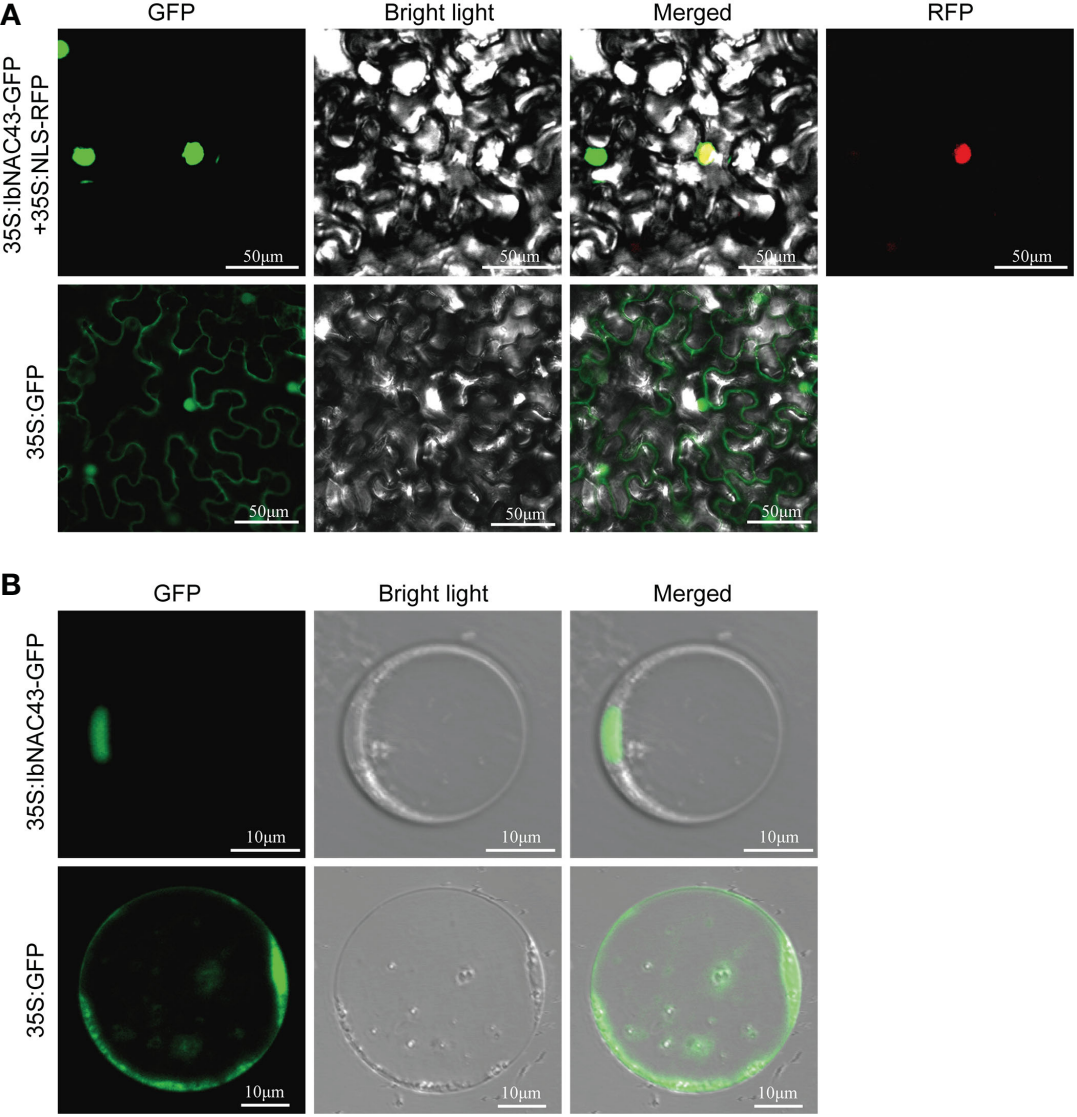
Subcellular localization of IbNAC43 in tobacco leaf epidermal cells **(A)** and rice protoplasts **(B)**. Confocal scanning microscopy images show that the 35S:IbNAC43-GFP fusion protein is localized in the nucleus, whereas the control 35S:GFP is not.

To study the subcellular localization of IbNAC43, the ORF of *IbNAC43* was fused with *GFP*, generating the fusion construct IbNAC43–GFP. The fusion construct and a nuclear localization protein, NLS-RFP, were then transiently co-expressed in *N. benthamiana* leaf epidermal cells using *A. tumefaciens*-mediated transformation. Microscopic visualization demonstrated that the fluorescence of the IbNAC43 fusion protein perfectly overlapped with that of NLS-RFP, indicating that IbNAC43 was localized in the nucleus (**Figure 3A**). We further analyzed the subcellular localization of IbNAC43 in rice protoplasts, and the result was consistent with the above-mentioned result (**Figure 3B**).

**Figure 4:**
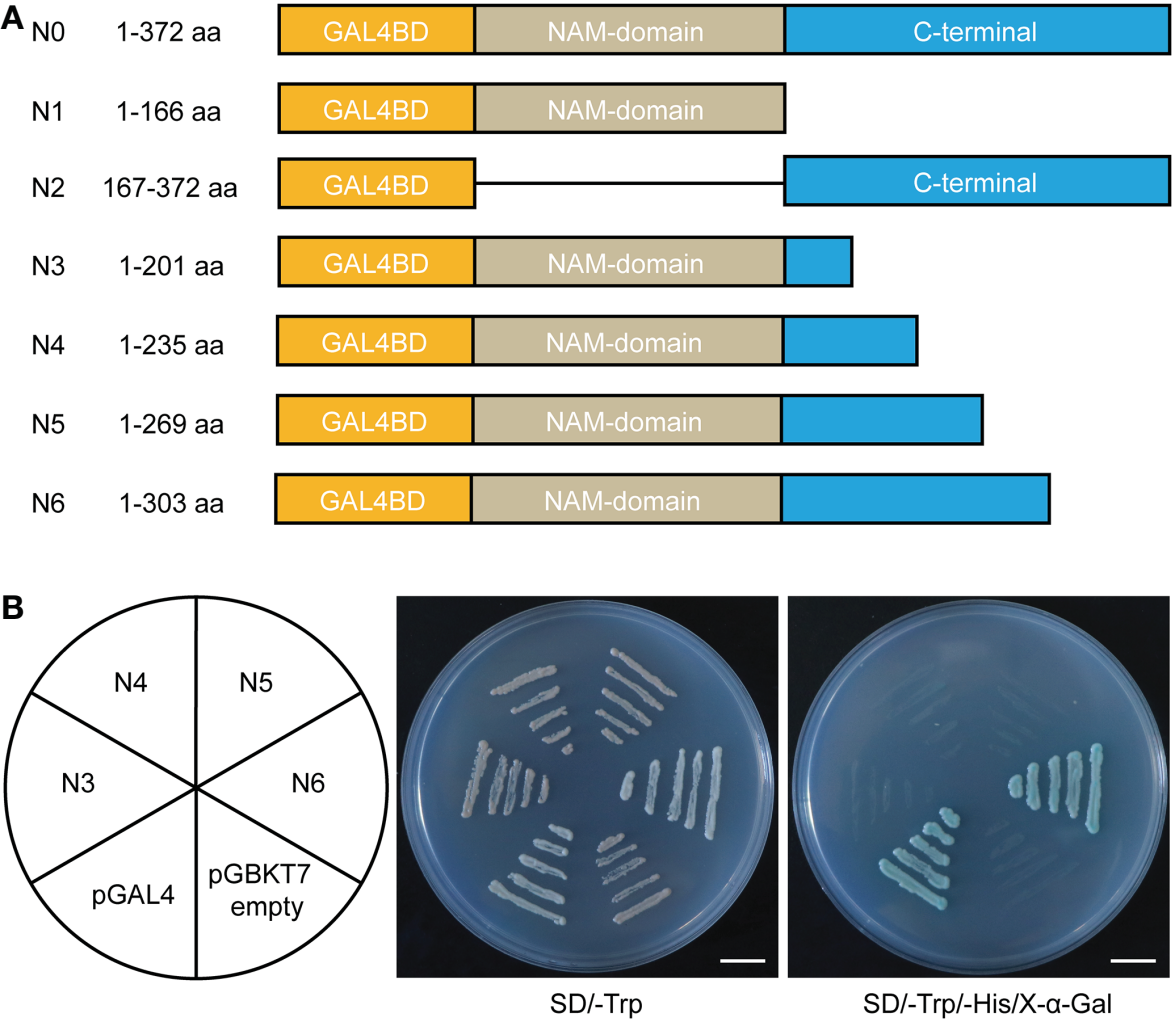
Transactivation activity assay of full-length and truncated IbNAC43 in yeast. **(A)** Schematic diagrams representing the IbNAC43 fragments encoding different parts of IbNAC43 that were cloned into the pGBKT7 vector. **(B)** Different fusion constructs were introduced into the yeast strain AH109 and examined on SD/-Trp and SD/-Trp/-His/X-α-Gal selection media. The pGBKT7 empty vector and pGAL4 were used as negative and positive controls, respectively. *Scale bar*, 1 cm.

To investigate the transactivation activity of IbNAC43, the different pGBKT7–IbNAC43 (1–372, 1–166, and 167–372 aa) fusion constructs and the pGBKT7 (negative control) and pGAL4 (positive control) vectors were separately transformed into the yeast strain AH109. Yeast cells containing any of the five vectors grew well on the SD/-Trp medium. On the other hand, yeast cells containing the N0 (1–372 aa), N2 (167–372 aa), and pGAL4 vectors grew well on the SD/-Trp/-His/X-α-Gal medium, while those containing the pGBKT7-IbNAC43 (1–166 aa) and pGBKT7 vectors did not grow (**Figure 4A**; **Supplementary Figure S2**). These results indicate that *IbNAC43* acted as a transcription activator and that the self-transcriptional activation domain was located in the C-terminal region. To determine whether any of the regions in IbNAC43 are responsible for TF activity, a series of fusion constructs, including N3 (1–201 aa), N4 (1–235 aa), N5 (1–269 aa), and N6 (1–303 aa) were transformed into yeast cells (**Figure 4A**). Results showed that yeast cells only harboring N6 or pGAL4 grew well on the SD/-Trp/-His/X-α-Gal medium (**Figure 4B**). The above results indicate that the region of 270–303 aa at the C-terminus was critical for the transactivation activity.

### Overexpression of *IbNAC43* causes leaf curling and plant dwarfism in sweet potato

**Figure 5:**
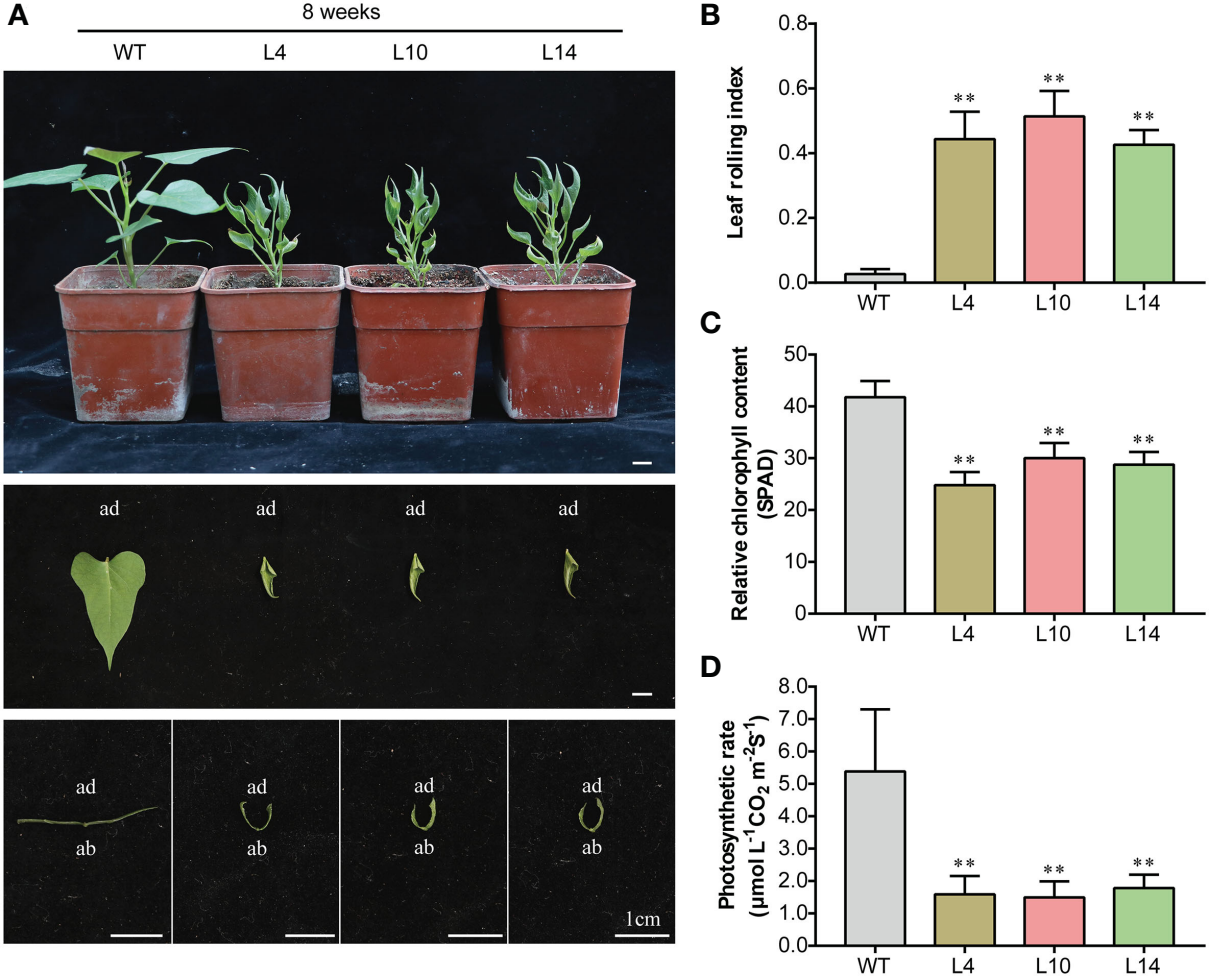
Phenotypes of transgenic and wild-type (WT) sweet potato plants in the greenhouse. **(A)** Plant stature and leaf phenotypes of both WT and transgenic plants. *Scale bar*, 1 cm. *ad*, adaxial side; *ab*, abaxial side. **(B)** Leaf rolling index (LRI). Data are presented as the mean ± SE (*n* = 6). **(C)** Relative chlorophyll content. **(D)** Photosynthetic rate. Data are presented as the mean ± SE (*n* = 3). *Asterisks* denote significant differences based on a Student’s *t*-test. **p < 0.01.

To evaluate the function of *IbNAC43*, this TF was transformed into the sweet potato cultivar Shangshu 19 using the *A. tumefaciens*-mediated transformation method. A total of six positive transgenic lines were obtained and confirmed by PCR. The qRT-PCR analysis showed that transgenic plants exhibited significantly higher expression levels of *IbNAC43* compared to WT plants (**Supplementary Figure S3**). The overexpression lines of *IbNAC43* showed a constitutively inward rolling phenotype from *in vitro* growth in MS medium to growth in the field (**Figure 5A**). In addition, we found that the degree of leaf curling increases with the growth of the plant. Compared to that in WT plants, the LRI in transgenic plants added up to ~0.25 at two weeks and to ~0.45 at eight weeks (**Figure 5B**; **Supplementary Figure S4**).

On the contrary, transgenic plants appeared dwarfed and showed growth retardation, with the size of their leaves being smaller compared to that of WT plants (**Supplementary Figure S3E**; **Figure 5A**); moreover, transgenic plants showed abnormal growth in the field (**Supplementary Figure S3I**). To elaborate on the reason for the dwarf phenotype, the analysis revealed that transgenic plants had a lower relative chlorophyll content and photosynthetic rate than WT plants (**Figures 5C, D**). These results indicate that the overexpression of *IbNAC43* caused leaf curling and dwarfism in transgenic sweet potato plants.

Subsequently, the expression of some cell cycle genes (e.g., *CYCA1;1*, *CYCA2;2*, *CYCB2;3*, and *CYCB3;1*) and cell expansion genes (e.g., *EXPA1*, *EXPA5*, and *EXPA15*) (Xu et al., 2016) was analyzed with qRT-PCR. The results showed that the cell cycle-related genes *CYCA1;1*, *CYCA2;2*, *CYCB2;3*, and *CYCB3;1* were downregulated in transgenic plants (**Supplementary Figure S5**). There were no significant differences between the cell expansion-related genes in WT and transgenic plants (**Supplementary Figure S5**). These results indicate that overexpression of *IbNAC43* might inhibit plant growth by negatively regulating a number of cell cycle genes.

### *IbNAC43* affects the balance of the adaxial and abaxial epidermis

**Figure 6:**
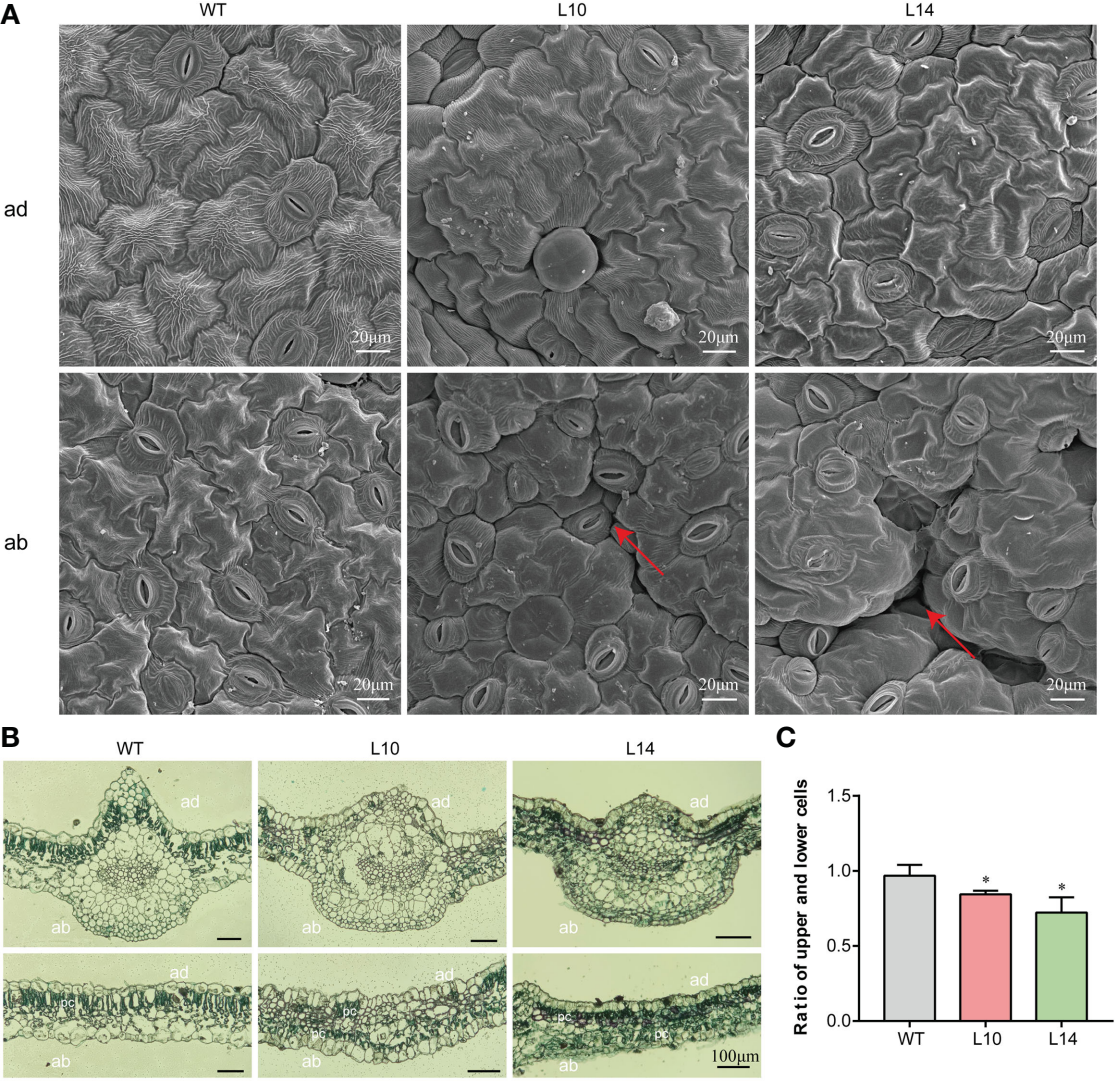
Cytological observations of the *IbNAC43*-overexpressed and wild-type (WT) plants. **(A)** The adaxial and abaxial sides of the leaf were subjected to SEM. *ad*, adaxial side; *ab*, abaxial side. *Scale bar*, 20 μm. **(B)** Paraffin sections of leaves. *pc*, palisade cells. *Scale bar*, 100 μm. **(C)** The ratio of the upper to lower epidermal cells. Data are values from three independent experiments. *Asterisk* indicates a significant difference compared to the WT based on a Student’s *t*-test. *p < 0.05.

As the morphological development of leaves depends on the morphology of the leaf epidermal cells, we thus compared the epidermal cells of transgenic and WT plants. The adaxial epidermal cells of the leaves were regular and uniform in both transgenic and WT plants. However, the size of the adaxial epidermal cells in WT plants was larger than that in transgenic plants (**Figure 6A**). In WT plants, the abaxial epidermal cells were regularly shaped and flat, whereas those of transgenic plants were irregular and uneven (**Figure 6A**). Thereafter, we further observed the cross-sections of the leaf blades in WT and transgenic plants. Compared to WT plants, transgenic plants displayed an interesting phenomenon in which the palisade mesophyll appeared to have a disorderly distribution (**Figure 6B**). These results indicate that the overexpression of *IbNAC43* caused leaf curling by affecting the ratio of adaxial to abaxial epidermal cells in transgenic sweet potato plants (**Figure 6C**).

### *IbNAC43* upregulates leaf adaxial polarity genes

**Figure 7:**
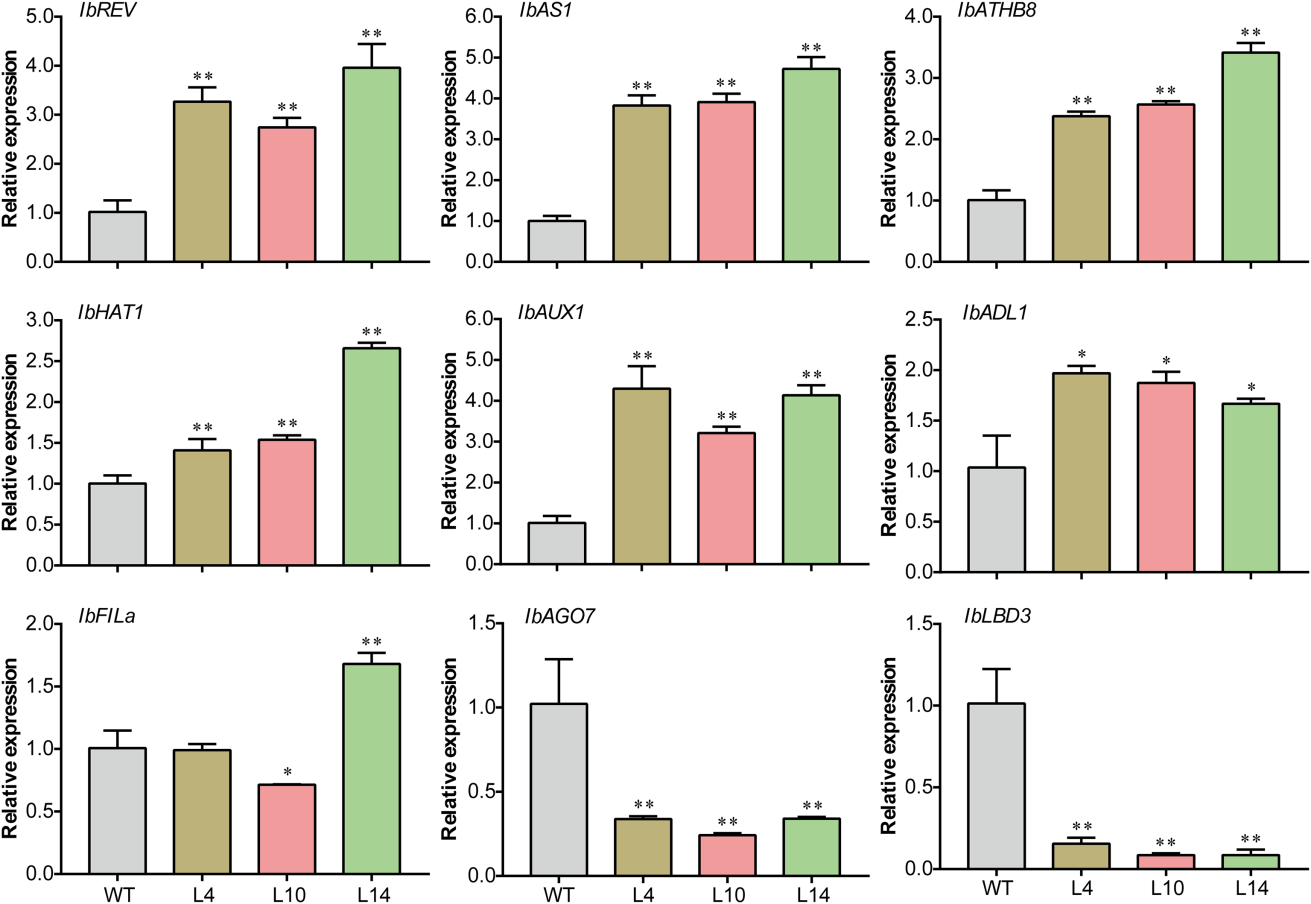
Expression of the genes associated with leaf polarity in both *IbNAC43*-overexpressed and wild-type (WT) sweet potato plants. Data are presented as the mean ± SE (*n* = 3). *Asterisks* indicate significant differences compared to the WT based on a Student’s *t*-test. *p < 0.05, **p < 0.01.

Previous reports showed that the establishment of leaf adaxial–abaxial polarity is associated with different genes in dicotyledonous plants, including the leaf adaxial (*REV*, *AS1*, *ATHB8*, and *HAT1*) and leaf abaxial (*FILa*) polarity genes. In addition, the plant hormone auxin was also found to cause leaf development defects, and *AUX1* was reported to influence leaf polarity by facilitating the movement of auxin from outside to inside the cells (Yang and Murphy, 2009). We examined the relative expression of these genes in WT and transgenic sweet potato plants. The adaxial-specific genes *IbREV*, *IbAS1*, *IbATH8*, and *IbHAT1* were significantly upregulated in the transgenic plants compared to WT plants (**Figure 7**). Specifically, the expression level of *IbAUX1* was significantly increased in transgenic plants (**Figure 7**).

Moreover, several genes that regulate leaf rolling have been identified in monocotyledonous plants such as rice (Sun et al., 2020; Xu et al., 2021). Genes homologous to the rice leaf rolling genes were detected in transgenic and WT plants, including *IbAGO7*, *IbLBD3*, and *IbADL1*. Compared to that in WT plants, the expression of *IbADL1* in transgenic plants was upregulated, whereas that of *IbAGO7* and *IbLBD3* was downregulated (**Figure 7**). These results indicate that *IbNAC43* might affect the development of leaves by regulating the expression of polarity-related genes.

### IbNAC43 participates in the establishment of leaf adaxial polarity by binding to the promoters of *IbREV* and *IbAS1*

**Figure 8:**
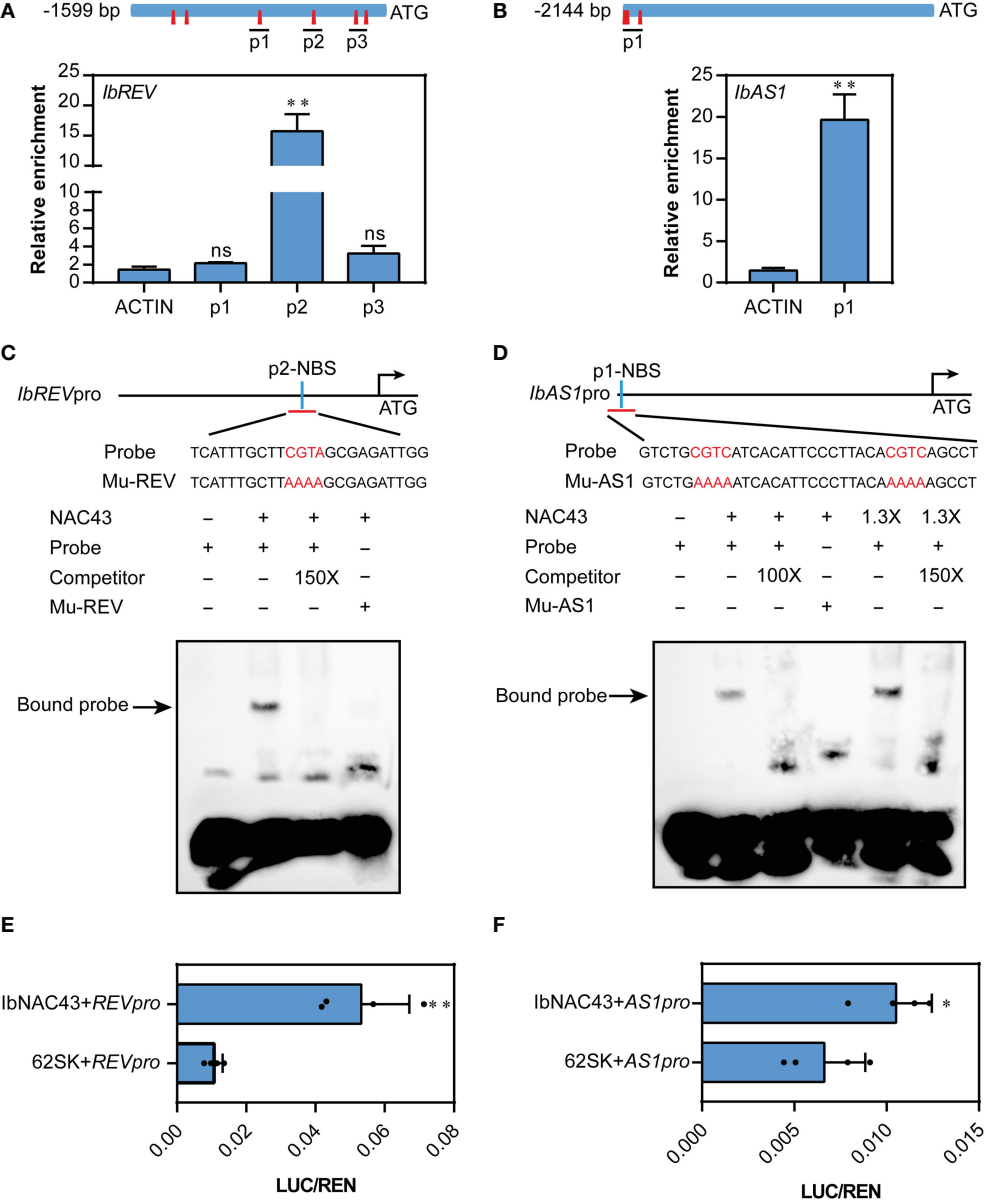
*IbNAC43* directly binds to the promoters of *IbREV* and *IbAS1*. **(A, B)** Locations of chromatin immunoprecipitation (ChIP)–quantitative PCR (qPCR) fragments and qPCR analysis of three biological replicates for the binding enrichment of *IbNAC43* in *IbREV* and *IbAS1* promoter regions. The PCR primers listed in **Supplementary Table S1** were designed to amplify the *IbREV* and *IbAS1* promoter regions. The NAC binding sequences (NBS) are represented by *red prisms*. **(C, D)** Electrophoretic mobility shift assays (EMSAs) demonstrate the interactions between *IbNAC43* and the DNA motifs. The recombinant protein 6×His-IbNAC43 retarded the shift of the probe. Both 100× and 150× unlabeled probes were used as competitors. The *plus sign* and *minus sign* indicate presence and absence, respectively. *NBS*, NAC binding sequences containing CGTG/A/C; *Mu-REV*, CGTA was mutated as AAAA; *Mu-AS1*, CGTG was mutated as AAAA. **(E, F)** *IbNAC43* triggered the activity of *IbREVpro*-LUC and *IbAS1pro*-LUC, as determined by the dual-luciferase assays in rice protoplasts. The expression level of Renilla (REN) was used as an internal control. Data are values from four independent experiments and are presented as the mean ± SE (*n* = 4). *Asterisks* indicate significant differences according to a Student’s *t*-test. *p < 0.05, **p < 0.01. ns, not significant.

NAC TFs function mainly by directly binding to the NAC binding sequences (NBS) containing CGTG/A/C and/or CACG (Nakashima et al., 2012; Kim et al., 2016; Diao et al., 2020). To investigate whether IbNAC43 can directly regulate the expression of the leaf adaxial polarity-related genes (i.e., *REV*, *AS1*, *ATHB8*, *AUX1*, and *HAT1*), we first established whether these genes contain the NBS in their promoter regions. It was found that the promoters of *REV* and *AS1* had different numbers of NBS elements (**Figures 8A, B**). This result indicates that IbNAC43 might bind to the promoters of *REV* and *AS1*. To confirm this possibility, we then performed the ChIP–qPCR assay using samples from OE-6 (overexpressed line 6). In this experiment, it was revealed that the association of IbNAC43 with the genomic regions of *REV* and *AS1* was significantly increased compared to the empty vector WT (**Figures 8A, B**).

Subsequently, we performed EMSA to further confirm the binding of IbNAC43 to these promoters *in vitro*. IbNAC43 bound directly to the *IbREV* or *IbAS1* promoter through the NBS motif, and binding was completed by excess unlabeled probes (**Figures 8C, D**). These results indicate that IbNAC43 directly binds to the promoters of *REV* and *AS1*.

In addition, we also examined whether IbNAC43 could directly regulate the expression levels of *REV* and *AS1* through transient dual-luciferase assays in protoplasts. LUC/REN levels were significantly increased when *IbREVpro*-LUC was co-transformed with 62SK-IbNAC43 and *IbAS1pro*-LUC was co-transformed with 62SK-IbNAC43 (**Figures 8E, F**), indicating that IbNAC43 could activate the expression levels of *IbREV* and *IbAS1*.

### Overexpression of *IbNAC43* promotes vascular bundle development

**Figure 9:**
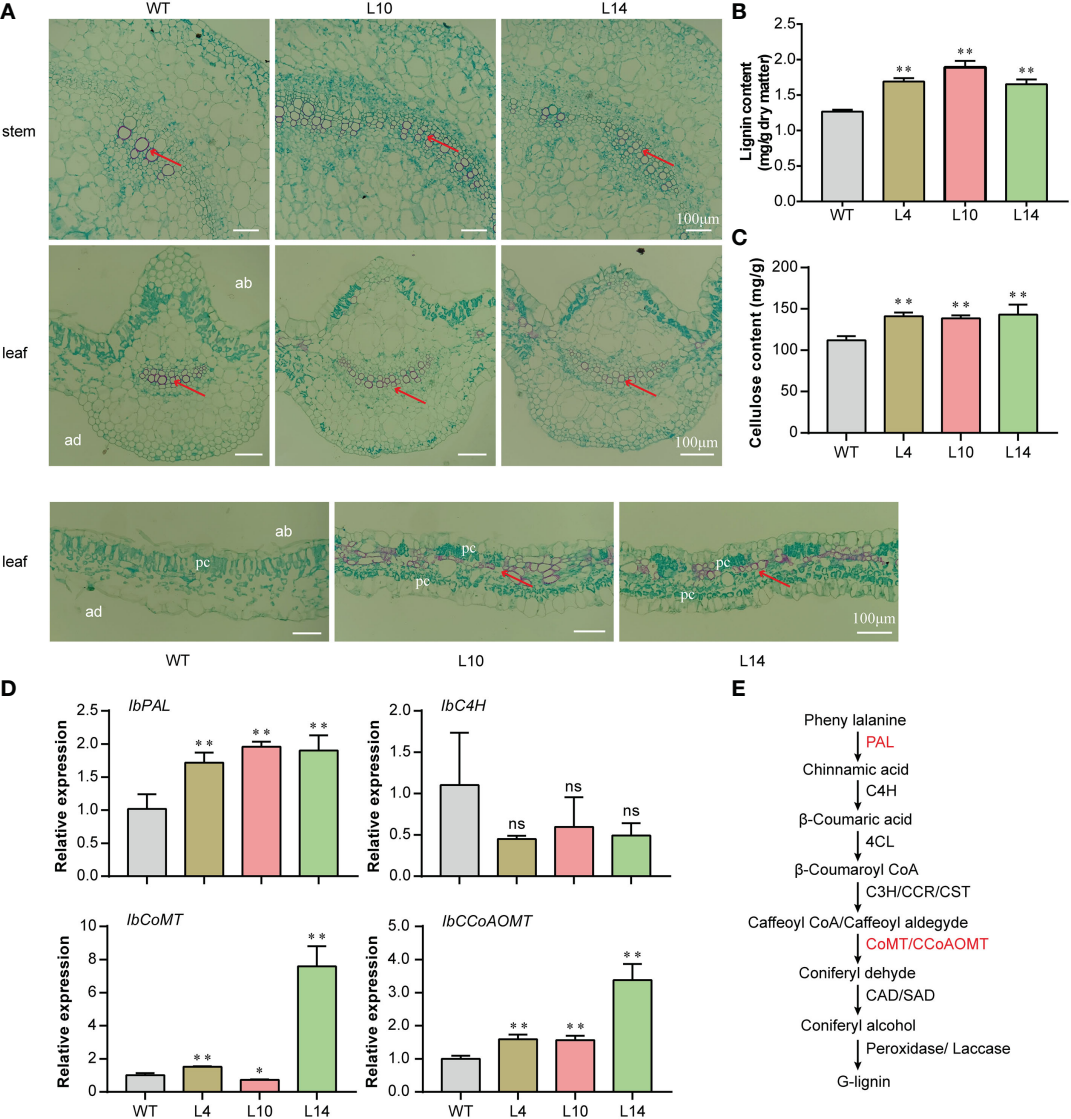
Histological observation of the *IbNAC43*-overexpressed and wild-type (WT) plants. **(A)** Paraffin sections. *ad*, adaxial side; *ab*, abaxial side; *pc*, palisade cells. *Scale bar*, 100 μm. **(B)** Lignin content. Data are presented as the mean ± SE (*n* = 3). **(C)** Mean cellulose content from *IbNAC43*-overexpressed and non-overexpressed sweet potato plants. Data are presented as the mean ± SE (*n* = 3). **(D)** Expression levels of the lignin biosynthesis-related genes. **(E)** Biosynthesis pathway of lignin. *PAL*, phenylalanine ammonia-lyase; *C4H*, cinnamate-4-hydroxylase; *4CL*, 4-coumarate-CoA ligase; *C3H*, *p*-coumaroyl-CoA3-hydroxylase; *CoMT*, caffeicacid-5-*O*-methyltransfenase; *CCoAOMT*, caffeoyl-CoA 5-*O*-methyltransfenase; *CAD*, cinnamyl alcohol dehydrogenase. *Asterisks* indicate significant differences compared to the WT based on a Student’s *t*-test. **p < 0.01. ns, not significant.

Previous studies showed that *ATHB8* can regulate leaf and vascular development and that its overexpression promotes vascular cell differentiation and lignin biosynthesis (Baima et al., 2001; Gardiner et al., 2011). In this study, the overexpression of *IbNAC43* upregulated the expression level of *IbATHB8*. We speculated that vascular cells might be affected in transgenic plants. Therefore, the paraffin sections of the leaves and stems were used for cytological observations. Stronger red staining was found in transgenic plants compared to WT plants (**Figure 9A**). The content of lignin in the leaves and stems was then determined. Compared to those of WT plants, the lignin and cellulose contents of transgenic plants were significantly increased (**Figures 9B, C**). The expression levels of the lignin synthesis-related genes (i.e., *IbPAL*, *IbCoMT*, and *IbCCoAOMT*) were significantly upregulated in the transgenic sweet potato plants compared to the WT (**Figures 9D, E**). We investigated whether *IbNAC43* can directly bind the promoters of *IbPAL*, *IbCoMT*, and *IbCCoAoMT*. Firstly, bioinformatics analysis revealed that the promoters of *IbPAL* and *IbCCoAoMT* had NBS motifs. Secondly, the Y1H assay indicated that IbNAC43 was unable to bind to the NBS of their promoters (**Supplementary Figure S6**). The above results suggest that the overexpression of *IbNAC43* promoted vascular bundle development in the leaves and stems of transgenic sweet potato plants.

## Discussion

The growth of plants depends on the normal development of their organs. Among the plant organs, the leaves play important roles in photosynthesis, yield, and abiotic stress response. The morphology of the leaves generally affects the photosynthetic efficiency and, consequently, plant growth. Inhibition of the expression of *DNL-4* reduced the plant height and leaf blade width and affected the productivity of transgenic rice plants (Bae et al., 2021). Leaf development originates from the shoot apical meristem (SAM), and the leaf primordium initiates growth and proliferates from the SAM. Adaxial–abaxial polarity is formed during this process. Its establishment is a complex process and has been revealed to be restricted *via* complicated regulatory networks (Du et al., 2018). However, there are no reports on the leaf development of sweet potato. In this study, we isolated the NAC TF *IbNAC43* from *I. trifida*, which is a wild ancestor of a sweet potato cultivar. *IbNAC43* was highly expressed in the leaves (**Figure 2**). Like most of the NAC proteins, IbNAC43 is a nuclear protein with self-transactivation activity in yeast (**Figure 3**). The overexpression of *IbNAC43* caused leaf curling and plant dwarfism, suggesting that it negatively regulated the growth of transgenic sweet potato plants (**Figure 5**).

The nutrients for plant growth and development come mainly from photosynthesis (Ramamoorthy et al., 2018). Chlorophyll is the most important plant pigment involved in photosynthesis. It absorbs and transmits light energy, initiates primary photochemical reactions, and directly affects photosynthetic efficiency (Tanaka and Tanaka, 2006; Zhao et al., 2019). An increased chlorophyll content enhances the photosynthetic efficiency, while its decrease reduces it (Chen and Xu, 2006). In general, enhanced photosynthesis can promote the growth and yield of plants. The overexpression of *SlSDH2.2* (succinate dehydrogenase gene) increased biomass and promoted growth by enhancing the rate of photosynthesis and reducing the rate of the tricarboxylic acid (TCA) cycle in transgenic tomato (Araujo et al., 2011). In contrast, reduced photosynthesis inhibits plant growth. *AtHXK1* encodes a hexokinase that senses the endogenous levels of sugars, and its overexpression reduced the chlorophyll content of leaves and the photosynthesis rates, inhibiting the growth of transgenic tomato (Dai et al., 1999). In this study, we found that the overexpression of *IbNAC43* decreased the photosynthesis rate by reducing the relative chlorophyll content in transgenic sweet potato plants (**Figure 5**).

Following the discovery of the first gene (*PHAN*) reported to influence leaf development, most of the TFs have been identified to serve important roles in partially redundant pathways. AS1 (an ARP MYB domain TF) and AS2 [a LATERAL ORGAN BOUNDARIES (LOB) domain gene] were found to directly bind to the promoters of *KNOX1* and *KNAT2* to repress their expression, which promoted leaf adaxial polarity formation (Guo et al., 2008). In addition, *REV*, *PHV*, *PHB*, *HAT1*, *ATHB4*, and *ATHB8*, which belong to the homeodomain leucine zipper (HD-ZIP) family, play positive roles in adaxial domain identification in *Arabidopsis*. However, the control of adaxial–abaxial leaf polarity is still complex, and the molecular regulatory mechanisms remain largely unknown. In this study, we found that the adaxial epidermal cells of WT plants were bigger than those of transgenic plants (**Figure 6A**), and the palisade mesophyll of transgenic plants appeared to have a disorderly distribution (**Figure 6B**). These results indicate that the overexpression of *IbNAC43* resulted in an imbalance in the number of upper and lower epidermal cells. qRT-PCR analysis showed that the overexpression of *IbNAC43* promoted leaf adaxial polarity by upregulating the expression levels of *REV*, *HAT1*, *ATHB8*, and *AS1* (**Figure 7**). In addition, our study revealed that IbNAC43 regulated the expression of *REV* and *AS1* by directly binding to their promoters (**Figure 8**).

In rice, a number of genes have been reported to regulate leaf rolling. Overexpression of *OsLBD3-7* and *OsAGO7* induced upward curling of the leaf blade (Shi et al., 2007; Li et al., 2016). The *adl1* mutant showed apparent adaxial leaves, indicating that *ADL1* has a negative role in adaxial leaf identification in rice (Hibara et al., 2009). Therefore, we further examined the expression levels of these homologous genes in transgenic sweet potato plants. Interestingly, in sweet potato, the overexpression of *IbNAC43* upregulated *IbADL1*, but downregulated *IbLBD3* and *IbAGO7* (**Figure 7**). These results indicate that similar/homologous genes in monocotyledons and dicotyledons might play different roles in leaf adaxial–abaxial polarity.

In general, the morphology and development of leaves are closely associated with lignin. Lignin is a complex organic polymer and is important for the structure and repair process of plant cell walls (Halpin, 2019). Proper lignin deposition in specialized leaf cells is important for leaf development (Yoon et al., 2015). The silencing of *PtrARF2.1* resulted in asymmetric leaf blades, reduced leaf area, lignin deposition, and a dwarf phenotype in transgenic *Paulownia tomentosa* plants (Fu et al., 2019). A lot of TFs have been reported to regulate lignin content and vascular bundle development. The NAC TFs *NST1* and *NST3* are key regulators of the formation of secondary walls and vascular vessels (Mitsuda et al., 2007). Extensive studies have found that the establishment of leaf polarity also affects the deposition of lignin. The *crm1-D* mutant (a gain-of-function mutant) showed leaf curling and a high lignin content, indicating that *OsRoc8* positively mediated lignin biosynthesis (Sun et al., 2020). Compared to the WT, the overexpression of *AtATHB8* caused plant dwarfism and showed a higher production of lignified tissue in transgenic plants (Baima et al., 2001). Moreover, *AtATHB8* can regulate leaf and vascular development and promote vascular cell differentiation (Gardiner et al., 2011). In this study, the overexpression of *IbNAC43* enhanced lignin biosynthesis and vascular bundle development by upregulating the expression of the lignin biosynthesis genes in transgenic sweet potato (**Figure 9**).

In conclusion, this is the first report on *IbNAC43* playing an important role in the establishment of leaf polarity and plant growth. The overexpression of this TF caused leaf curling, reduced the photosynthetic rate, and retarded plant growth by upregulating the leaf adaxial polarity-related genes. Furthermore, we identified a new regulatory mechanism in IbNAC43, which promotes the transcription of *IbREV* and *IbAS1* by directly binding to their promoters. This study provides additional insights into understanding the effects of leaf adaxial–abaxial development on plant growth in dicotyledons.

## Data availability statement

The raw data supporting the conclusions of this article will be made available by the authors, without undue reservation, to any qualified researcher.

## Author contributions

SS and HoZ: Conceptualization. XL and NN: Data curation. XL and YC: Formal analysis. SS and HoZ: Writing-original draft. SG, HuZ, SH, QL, and HoZ: Writing-review and editing. All authors contributed to the article and approved the submitted version.
