# Supplementary material for: Sweet potato NAC transcription factor NAC43 negatively regulates plant growth by causing leaf curling and reducing photosynthetic efficiency
Source: Front Plant Sci. 2023 Feb 21;14:1095977. doi: 10.3389/fpls.2023.1095977 (PMC9988925; doi:10.3389/fpls.2023.1095977)
Supplement: Supplementary file 1 [file DataSheet_1.docx]

**Supplemental Figure S1.** Sequence analysis of NAC43 in wild and cultivated sweetpotato (*I. trifida* and Shangshu 19). (A) Multiple sequence alignment of the IbNAC43 protein in *I. trifida* and Shangshu 19. (B) The genomic structures of *IbNAC43* in *I. trifida* and Shangshu 19. IbNAC43-W: the IbNAC43 protein in *I. trifida*; IbNAC43-S19: the IbNAC43 protein in Shangshu 19. Exons are represented by boxes, and introns are represented by lines.

**Supplemental Fig. S2.** The different fusion constructs were transformed into the yeast strain AH109 and examined on SD/-Trp and SD/-Trp/-His/X-α-Gal selection medium. The pGBKT7 empty vector and pGAL4 were used as negative and positive controls, respectively (Scale bars = 1 cm).

**Supplemental** **Fig. S3.** Production of transgenic sweetpotato plants overexpressing the *IbNAC43* gene. (A) Embryogenic calluses. (B) Embryogenic suspension cultures of sweetpotato cv. Shangshu 19. (C) Formation of Hyg-resistant embryogenic calluses. (D) Germination of somatic embryos. (E) Regeneration of plantlets. (F) PCR analysis of the *IbNAC43*-overexpression plants. (G) Expression levels of *IbNAC43* in transgenic plants and WT. (H) WT grown in field. (I) Transgenic plants grown in field. ** indicates a significant difference compared to the WT at *p* < 0.01 based on Student’s *t*-test, respectively.

**Supplemental** **Fig. S4.** The leaf morphology of the *IbNAC43*-overexpression plants and WT after 2 weeks. (A) Phenotypes, ad: adaxial side; ab: abaxial side. (B) Leaf rolling index (LRI). Data are presented as means ± SE (*n* = 3). Asterisks denote significant differences based on Student’s *t*-test (** *p* < 0.01). Scale bars = 1 cm.


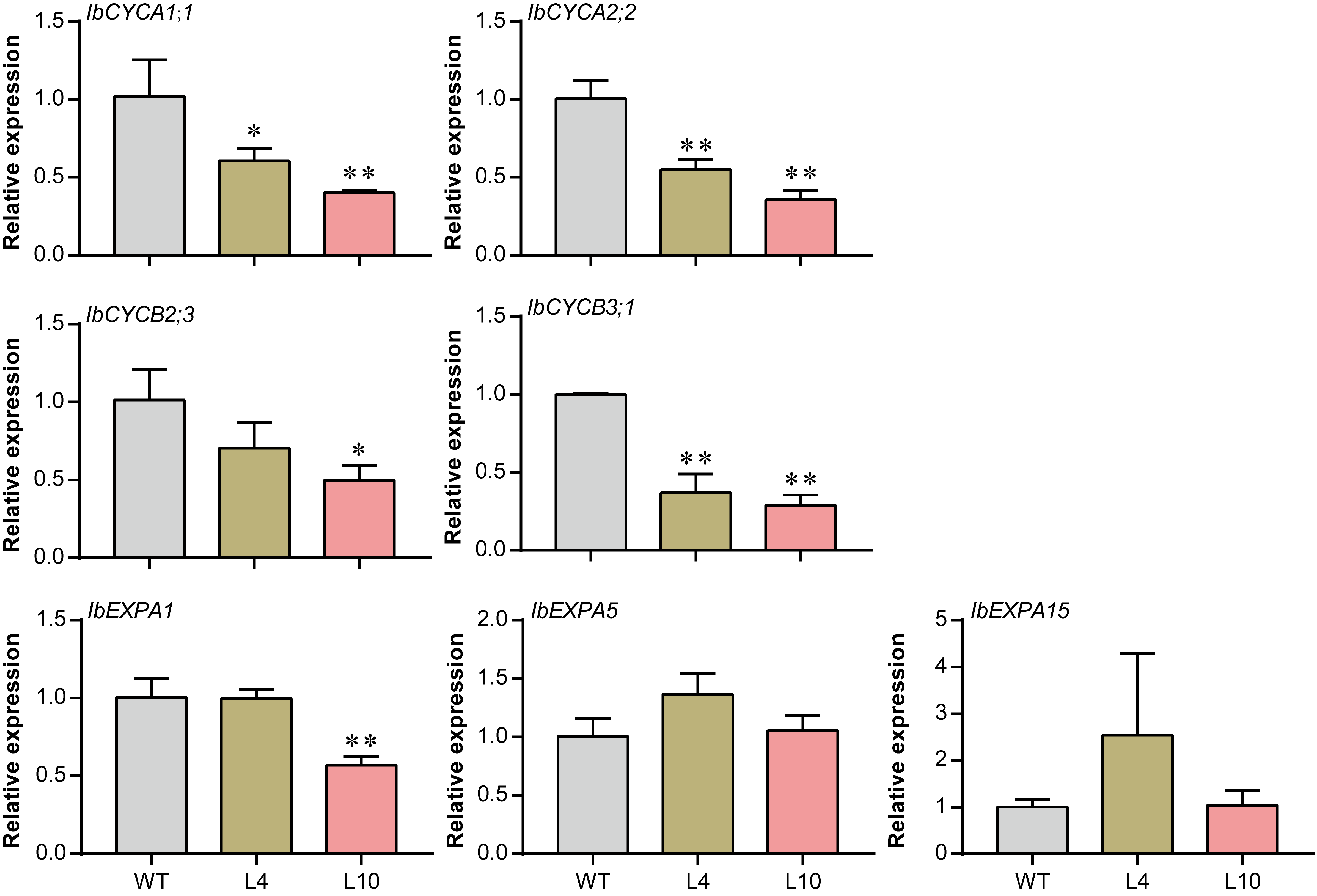


**Supplemental Fig. S5.** Expression analysis of the genes associated with cell cycle-related genes and cell expansion-related genes in the *IbNAC43*-overexpression sweet potato plants and WT. Data are presented as the means ± SE (*n* = 3). * and ** indicate a significant difference compared to the WT at *p* < 0.05 and *p* < 0.01 based on Student’s *t*-test, respectively.


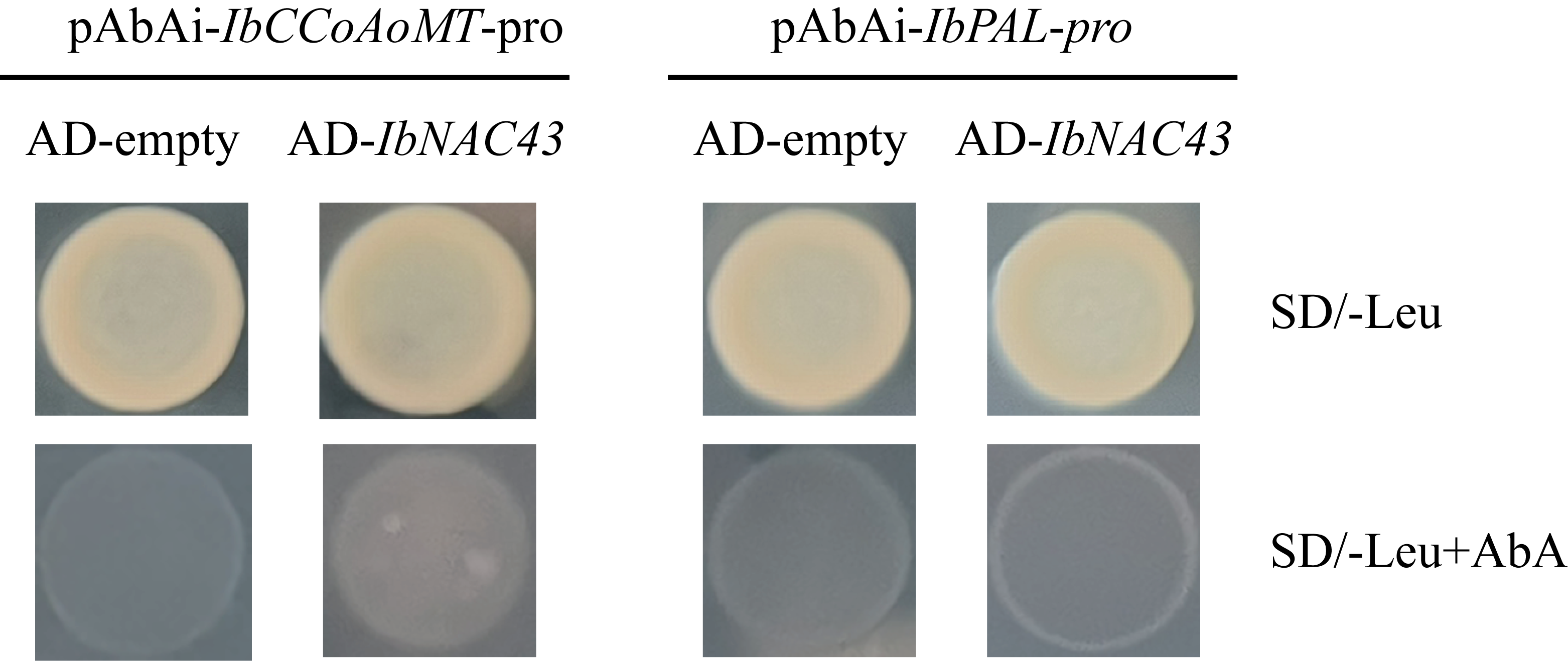


**Supplemental Fig. S6.** Y1H assay on binding of IbNAC43 protein to the promoters of *IbCCoAoMT* and *IbPAL*. The sequences containing the NBS motif were cloned into pAbAi plasmid. Negative control: pGADT7+pAbAi-*IbCCoAoMT*-pro; pGADT7+pAbAi-*IbPAL*-pro. The transformed yeast cells were incubated in the plate lacking SD/-Leu with or without AbA. And the AbA concentration was 300 ng/mL.
