# Supplementary material for: Sweet potato NAC transcription factor NAC43 negatively regulates plant growth by causing leaf curling and reducing photosynthetic efficiency
Source: Front Plant Sci. 2023 Feb 21;14:1095977. doi: 10.3389/fpls.2023.1095977 (PMC9988925; doi:10.3389/fpls.2023.1095977)
Supplement: Supplementary file 2 [file DataSheet_2.docx]

**Supplementary Table S1**. Primers used in this study.

| Primer name | Primer sequence (5′-3′) |
| --- | --- |
| Primers for ORF/genome DNA |  |
| *IbNAC43*-ORF-F | ATGAATCTTTCCGTGAATGGTC |
| *IbNAC43*-ORF-R | CTATACCGACAAGTGGGACAAGG |
| Primers for promoter of *IbNAC43* |  |
| *IbNAC43*-promoter-F | TCGCCGATCCGACTACTAAA |
| *IbNAC43*-promoter-R | CTTGCACTTCTCTTGAATATCCC |
| Primers for overexpression vector/subcellular localization |  |
| 1300-*IbNAC43*-F | ACGGGGGACGAGCTCGGTACCATGAATCTTTCCGTGAATGGT |
| 1300-*IbNAC43*-R | GCTCACCATGTCGACTCTAGATACCGACAAGTGGGACAAGG |
| Primers for identifying overexpression plants |  |
| 35s-F | TCCTTCGCAAGACCCTTCCTC |
| JD-IbNAC43-R | TACCGACAAGTGGGACAAGG |
| Primers for qRT-PCR |  |
| qRT-IbFILa-1-f | GCCTGATCAACCCGTGAAGA |
| qRT-IbFILa-1-r | TGGTTGTGCCAGAAACCCAT |
| qRT-IbADL1-f | CGAGTCCAGGATGAGGTTCG |
| qRT-IbADL1-r | AACCGCCTACGTTCTTCTGG |
| qRT-IbREV-f | CCGGGAATGAGGCCTACAAG |
| qRT-IbREV-r | TCGTTGTCGAGAGCATGTCC |
| qRT-IbATHB8-f | GGTCATGCCTTTTCTCCCGA |
| qRT-IbATHB8-r | CTCCCGCAGCATTCTCATCA |
| qRT-IbHAT1-f | CAGCATCGTCTCCGGCAATA |
| qRT-IbHAT1-r | AGGGTCTACACAGGTTCCCA |
| qRT-IbATHB4-f | AAGAAAACGATGCGGAACGG |
| qRT-IbATHB4-r | AGCTCTCTTCCAGAATGGCG |
| qRT-IbAUX1-f | GACCAGCTTCTCGACCACTC |
| qRT-IbAUX1-r | ACGCGAACCCGAACGTAATA |
| qRT-IbRoc8-f | TACGCGCCACCTTACAGAAG |
| qRT-IbRoc8-r | ATTGGGAGCCAAATGGTGGT |
| qRT-IbLBD3-f | GCTGGCGATTACACAAGCTG |
| qRT-IbLBD3-r | CCGCCGTGAGTAGTATGAGC |
| qRT-IbNAC43-F | GAACCGTCCTGCAACAACAC |
| qRT-IbNAC43-R | GAGGATTCGATCTGGCCGTT |
| qRT-*Actin*-F | AGCAGCATGAAGATTAAGGTTGTAGCAC |
| qRT-*Actin*-R | TGGAAAATTAGAAGCACTTCCTGTGAAC |
| qRT-IbPAL-F | CCATTCCCAACAGAATCACC |
| qRT-IbPAL-R | CTTGAGGCATCTCAACAATG |
| qRT-IbC4H-F | GGTCGTAGTGTCGAACCCTG |
| qRT-IbC4H-R | TGAACACCATGTCCTGACCG |
| qRT-Ib4CL-F | TGGGTTCCACCGGAAAAACA |
| qRT-Ib4CL-R | CCGATCATAGAAGCCGCCAT |
| qRT-IbCOMT-F | ACAAAATGGGTTCGACCGGA |
| qRT-IbCOMT-R | CCATAGGGAGAACAGAGGCG |
| qRT-IbCCoAOMT-F | TTTCATTTTCGTGGACGCCG |
| qRT-IbCCoAOMT-R | GTTCCAGAGGGTGTTGTCGT |
| qRT-IbCYCA1.1-F | GGAAAATCGCCACGGAAAGG |
| qRT-IbCYCA1.1-R | GCTTCGCGATGAAAGCAGAG |
| qRT-IbCYCA2.2-F | GAGGCCTACCCCCTGTAAAC |
| qRT-IbCYCA2.2-R | CTGACCAGAAGTGGCACCAG |
| qRT-IbCYCB2.3-F | AACTAATGCTCAAGGTGGGGG |
| qRT-IbCYCB2.3-R | CTCCCAGTGCTCTCCTGTTG |
| qRT-IbCYCB3.1-F | GCAAGACCGTATACCTGGCA |
| qRT-IbCYCB3.1-R | TGCTGAGAGAAATGCGTCCT |
| qRT-IbEXPA1-F | CGTCATTTTCGTGGGGCTTG |
| qRT-IbEXPA1-R | CCCACAGCTCAACCCATTGT |
| qRT-IbEXPA5-F | ACACTGCTGCACTTAGCACA |
| qRT-IbEXPA5-R | AGGACCGATCGTTCACACAC |
| qRT-IbEXPA15-F | CGATGGAAAATGGTGCGGTC |
| qRT-IbEXPA15-R | ATGGAACCCTGCGATAACGG |
| Primers for transactivation activity |  |
| BD-N43-1-F | ATCTCAGAGGAGGACCTGCATATGATGAATCTTTCCGTGAATGGT |
| BD-N43-1-R | GTCGACGGATCCCCGGGAATTCGCAGACCACCCAACCTTCT |
| BD-N43-2-F | ATCTCAGAGGAGGACCTGCATATGCGTGTCTTCAAGAAGAAAAACTAT |
| BD-N43-2-R | GTCGACGGATCCCCGGGAATTCCTATACCGACAAGTGGGACAAG |
| BD-N43-3-R | GTCGACGGATCCCCGGGAATTCGTAAGTAAGGATTTGATCCAGAACG |
| BD-N43-4-R | GTCGACGGATCCCCGGGAATTCGATTGCATGGGAGAATTGCA |
| BD-N43-5-R | GTCGACGGATCCCCGGGAATTCTTCGGCCAGCATGTCTTC |
| BD-N43-6-R | GTCGACGGATCCCCGGGAATTCGAGCTGAGAGGCGACGAG |
| Primers for ChIP-qPCR |  |
| AS1pro-p1-F | CTGGGTCTGCGTCATCACAT |
| AS1pro-p1-R | TCTTTGTAGCGGGGAAGTGT |
| REVpro-p1-F | GACGACGCATCACTCTCTCC |
| REVpro-p1-R | CAGCAAGAAACGGAAACCCTC |
| REVpro-p2-F | GAGCCGGAGCTCAACTCATT |
| REVpro-p2-R | AAACAACTCGTGCCAAACGG |
| REVpro-p3-F | AAGGCAAGCCTGAAGTGTGT |
| REVpro-p3-R | AAGCGGAAGATTCTTGGGTCC |
| *β*-*actin*-F | ACCTGCGAGACCCACTTATT |
| *β*-*actin*-R | ACAAGCTAGTCCTAACCCACC |
| Primers for EMSA assay |  |
| pET28a-IbN43-F | TGACTGGTGGACAGCAAATGGGTCGCGGATCCATGAATCTTTCCGTGAATGGT |
| pET28a-IbN43-R | GCGGCCGCAAGCTTGTCGACGGAGCTCGAATTTACCGACAAGTGGGACAAGG |
| proAS1-BJ-F (5'-Biotin) | GTCTGCGTCATCACATTCCCTTACACGTCAGCCT |
| proAS1-BJ-R (5'-Biotin) | AGGCTGACGTGTAAGGGAATGTGATGACGCAGAC |
| proAS1-JZ-F | GTCTGCGTCATCACATTCCCTTACACGTCAGCCT |
| proAS1-JZ-R | AGGCTGACGTGTAAGGGAATGTGATGACGCAGAC |
| proAS1-mBJ-F(5'-Biotin) | GTCTGAAAAATCACATTCCCTTACAAAAAAGCCT |
| proAS1-mBJ-R(5'-Biotin) | AGGCTTTTTTGTAAGGGAATGTGATTTTTCAGAC |
| proREV-BJ-F (5'-Biotin) | TCATTTGCTTCGTAGCGAGATTGG |
| proREV-BJ-R (5'-Biotin) | CCAATCTCGCTACGAAGCAAATGA |
| proREV-JZ-F | TCATTTGCTTCGTAGCGAGATTGG |
| proREV-JZ-R | CCAATCTCGCTACGAAGCAAATGA |
| proREV-mBJ-F(5'-Biotin) | TCATTTGCTTAAAAGCGAGATTGG |
| proREV-mBJ-R(5'-Biotin) | CCAATCTCGCTTTTAAGCAAATGA |
| Primers for dual-luciferase assays |  |
| 62sk-IbN43-F | AGTGGATCCCCCGGGCTGCAGATGAATCTTTCCGTGAATGGTC |
| 62sk-IbN43-R | TGATTTCAGCGAATTGGTACCCTATACCGACAAGTGGGACAAGG |
| 0800-AS1-P-F | CTATAGGGCGAATTGGGTACCTCTGGGTCTGCGTCATCAC |
| 0800-AS1-P-R | AGTGGATCCCCCGGGCTGCAGCACAAACAGCAGGCAGGTAAT |
| 0800-REV-P-F | CTATAGGGCGAATTGGGTACCCCCCATACAAGAACAAGACGA |
| 0800-REV-P-R | AGTGGATCCCCCGGGCTGCAGGGATCTTGATTGAAGCGGAAG |
| Primers for yeast one hybrid |  |
| AD-NAC43-F | GTACCAGATTACGCTCATATGATGAATCTTTCCGTGAATGGTC |
| AD-NAC43-R | ATGCCCACCCGGGTGGAATTCCTATACCGACAAGTGGGACAAGG |
| pAbAi-CCoAoMT-F | GAATTCGAGCTCGGTACCGAGTCCATAGCCTTAACAAGAA |
| pAbAi-CCoAoMT-R | CAGAGCACATGCCTCGAGATAATAGGCAAGGGAAGGGAAG |
| pAbAi-PAL-F | GAATTCGAGCTCGGTACCTCCTCCAATTCATTGTCGTTAT |
| pAbAi-PAL-R | CAGAGCACATGCCTCGAGCAACAGGCATGATTTGTACTCG |

**Supplementary Table S2**. *Cis*-acting elements in the promoter of *IbNAC43*.

| regulatory elements | numbers of element | position (-ATG) | function |
| --- | --- | --- | --- |
| ABRE | 1 | -502 bp | Involved in the abscisic acid responsiveness |
| G-box | 2 | -502 bp, -506 bp | Involved in light responsiveness |
| GARE-motif | 1 | -411 bp | Gibberellin-responsive element |
| MYB | 4 | -362 bp, -410 bp, -418 bp, -1594 bp | MYB transcription factors binding element |
| MYC | 2 | -1533 bp, -1386 bp | Involved in abiotic stress |
| TCCC-motif | 1 | -218 bp | Light responsive element |
| TCT-motif | 2 | -1095 bp, -1260 bp | Light responsive element |
